# Ca^2+^ sensitivity changes in skinned myocardial fibers induced by myosin–actin crossbridge-independent sarcomere stretch: Role of N-domain of MyBP-C

**DOI:** 10.1016/j.yjmcc.2025.03.004

**Published:** 2025-03-09

**Authors:** Xutu Wang, Nathan Kallish, R. John Solaro, Wen-Ji Dong

**Affiliations:** a Voiland School of Chemical Engineering and Bioengineering, Washington State University, Pullman, WA 99163-1062, USA; b Department of Physiology and Biophysics, College of Medicine, University of Illinois at Chicago, Chicago, IL 60612-7342, USA; c Department of Integrative Physiology and Neuroscience, Washington State University, Pullman, WA 99163-1062, USA

**Keywords:** Length-dependent activation, Myosin-binding protein C, N-terminal region, C0-C2 calcium sensitivity, Mavacamten, Transgenic rat

## Abstract

Sarcomere length-dependent activation (LDA) is the key cellular mechanism underlying the Frank-Starling law of the heart, in which sarcomere stretch leads to increased Ca^2+^ sensitivity of myofilament and force of contraction. Despite its key role in both normal and pathological states, the precise mechanisms underlying LDA remain unclear but are thought to involve multiple interactions among sarcomere proteins, including troponin of the thin filament, myosin, titin and myosin binding protein C (MyBP-C). Our previous study with permeabilized rat cardiac fibers demonstrated that the mechanism underlying the increase in Ca^2+^ sensitivity of thin filament induced by sarcomere stretch may involve sarcomere length (SL)-induced interactions between troponin and weakly bound, disordered relaxed state (DRX) myosin heads in diastole, rather than strong myosin–actin crossbridge interactions. In this study we investigated the role of the N-domains of MyBP-C in this newly discovered mechanism. To examine the potential role of the N-domain of MyBP-C in SL-induced myosin-troponin interactions, skinned myocardial fibers from a transgenic ΔN-MyBP-C rat with deleted N-terminal C0-C2 domains and a non-transgenic rat were reconstituted with troponin containing wild-type cTnT, cTnC(13C/51C)_AEDANS-DDPM_ and mutant ΔSP-cTnI or wild-type cTnI. Because the switching peptide (SP) of ΔS-cTnI is replaced by a nonfunctional peptide linker, force-generating actin-myosin crossbridge interactions of the reconstituted skinned fibers with mutant ΔSP-cTnI are inhibited regardless of the presence of Ca^2+^. This approach allowed us to examine the sensitivity of troponin/thin filament to Ca^2+^ binding in response to sarcomere stretch by monitoring Ca^2+^-induced changes in fluorescence resonance energy transfer (FRET) between AEDANS and DDPM attached to the N-domain of cTnC in the presence/absence of myosin–actin crossbridge interaction with or without deletion of C0-C2 domains of MyBP-C. Our measurements of SL-induced changes in muscle fiber mechanics and FRET Ca^2+^ sensitivities provide strong evidence that both the weakly bound myosin heads and the N-terminus of MyBP-C are critical for SL to activate troponin in the diastolic state. A model based on the results is proposed for the mechanism underlying LDA of myofilament.

## 1. Introduction

In healthy hearts, as the myocardium is stretched due to an increase in venous return, myofilaments become more sensitive to Ca^2+^. This phenomenon is known as length-dependent activation (LDA) and is the cellular basis of the Frank-Starling (FS) law of the heart, a mechanism in which the heart increases its stroke volume in response to an increase in venous return [1]. The physiological significance of LDA in cardiac muscle is highlighted by the observation that LDA is impaired in heart failure patients [2–4], and evidence suggests that attenuation of LDA at the myofilament level may translate to an impaired FS mechanism in the intact heart [5–7]. Despite its clinical and physiological significance, the precise mechanisms underlying LDA remain unclear but are thought to involve multiple interactions among sarcomere proteins, including the troponin of the thin filament, myosin, titin and myosin binding proteins C (MyBP-C) [1,8–10].

Current consensus is that the sarcomere stretch-induced increase in myofilament Ca^2+^ sensitivity is related to structural changes in the thin filament [11–13] caused by a feedback pathway through strongly bound myosin–actin crossbridges (XBs) [14–17]. However, in a recent study (the 1st manuscript), we showed evidence that the increase in Ca^2+^ sensitivity induced by sarcomere stretch is independent of strong myosin–actin XBs but requires myosin heads in a weakly bound (DRX) state. That study was performed with a platform of reconstituted skinned myocardial fibers in which the native troponin was replaced with a troponin complex containing a fluorophore-modified troponin C, cTnC (13C/51C)_AEDANS-DDPM_, and a recombinant troponin I mutant, ΔSP-cTnI, in which the switching peptide (Sp) of cTnI was replaced with a nonfunctional peptide link to block actin–myosin XB interactions. This platform allows the simultaneous measurement of mechanics of the reconstituted skinned fibers and the Ca^2+^ sensitivity of troponin by monitoring changes in fluorescence resonance energy transfer (FRET) between donor AEDENS and acceptor DDPM attached to the N-domain of cTnC. Muscle mechanics and FRET fluorescence intensity measurements made with this platform clearly showed that sarcomere stretch induced the same increases in troponin Ca^2+^ sensitivity in either the presence or the absence of strong XBs, suggesting that the SL-induced change in Ca^2+^sensitivity is independent of strong myosin–actin XBs. Further study with skinned myocardial fibers reconstituted with troponin containing engineered TEV-digestible mutant cTnI and cTnT as well as mavacamten, a selective myosin-motor inhibitor, strongly suggested that SL-induced interaction between troponin and weakly bound myosin in diastole, rather than strong myosin–actin XBs, is the molecular mechanism underlying LDA of myofilament. Previous studies reported that associated with the presence of XBs like DRX motors in the vicinity of thin filaments is a binding of the N-domain of cardiac MyBP-C to the thin filament [18]. This binding had been reported to sensitize the thin filaments to Ca^2+^ [19]. In this study, we used the same platform described above to investigate the role of the N-domain of cardiac MyBP-C in the SL-induced change in troponin Ca^2+^ sensitivity caused by the proposed myosin–troponin interaction.

There is ample evidence that in addition to troponin/thin filament, myosin filament and titin, MyBP-C is another important component of myofilaments that participates in muscle regulation, including LDA [8,10,20–23]. The importance of MyBP-C in heart function is highlighted by the discovery of more than 200 heritable mutations in the human MYBP-C3 gene. These mutations have been linked to cardiomyopathy, heart failure, sudden cardiac death [24,25], and attenuated LDA in human heart failure [24,26]. Cardiac MyBP-C is a 140-kDa protein in a long rod shape that is 40 nm long and 3 nm thick and is known to be structurally confined to one half the length of the thick filament in a pattern that suggests the MyBP-C interacts with only every third level of myosin molecules [27–29]. Studies with MyBP-C knockout mouse hearts revealed that MyBP-C normally constrains the interaction of myosin heads with actin and that ablation of MyBP-C leads to an acceleration of XB cycling [30–33] as well as blunting SL-dependent changes in the contractile dynamics of muscle [34]. Although results indicate that the role of MyBP-C in LDA involves dynamic interactions of its N-terminal C0-C1 region with both the S2 region of myosin in thick filament and actin in TF [35–39], the exact mechanistic role in LDA is still elusive [40]. In this study, skinned myocardial fibers from wild-type rats and a transgenic (TG) rat model, ΔN-MyBP-C, with the N-terminal C0-C2 deleted, were used to investigate the potential effects of the C0-C2 domains of MyBP-C on the myosin-actin XB-independent SL-induced changes in the Ca^2+^ sensitivity of troponin observed in our previous study (the 1st manuscript) in the presence and the absence of strong myosin actin XBs. As in our previous study (the the 1st manuscript), to block the strong myosin–actin XB formation, the native cTnI of the skinned fibers was replaced by ΔSP-cTnI mutant. Our measurements of SL-induced changes in muscle fiber mechanics and FRET Ca^2+^ sensitivities provide strong evidence that both the weakly bound myosin heads and the N-terminus of MyBP-C are critical for sarcomere stretch to activate troponin in the diastolic state, *i.e*., in the absence of Ca^2+^ thin filament activation and myosin–actin XB.

## 2. Results

### 2.1. Verification of the C0-C2 deletion in ΔN-MyBP-C rats

To examine the effect of the N-terminal C0 to C2 region of MyPB-C on myofilament regulation, a genetically engineered ΔN-MyBP-C rat model with the C0-C2 domains of MyBP-C deleted was developed. Since this is a non-physiological and non-pathological rat model and is specifically designed as a template tool for our current project to examine potential roles of each of the C0-C2 domains in myofilament regulation, full assessment of phenotype of this rat model was not performed at in this study. However, this assessment will be carried out in future study.

**Fig. 1.**
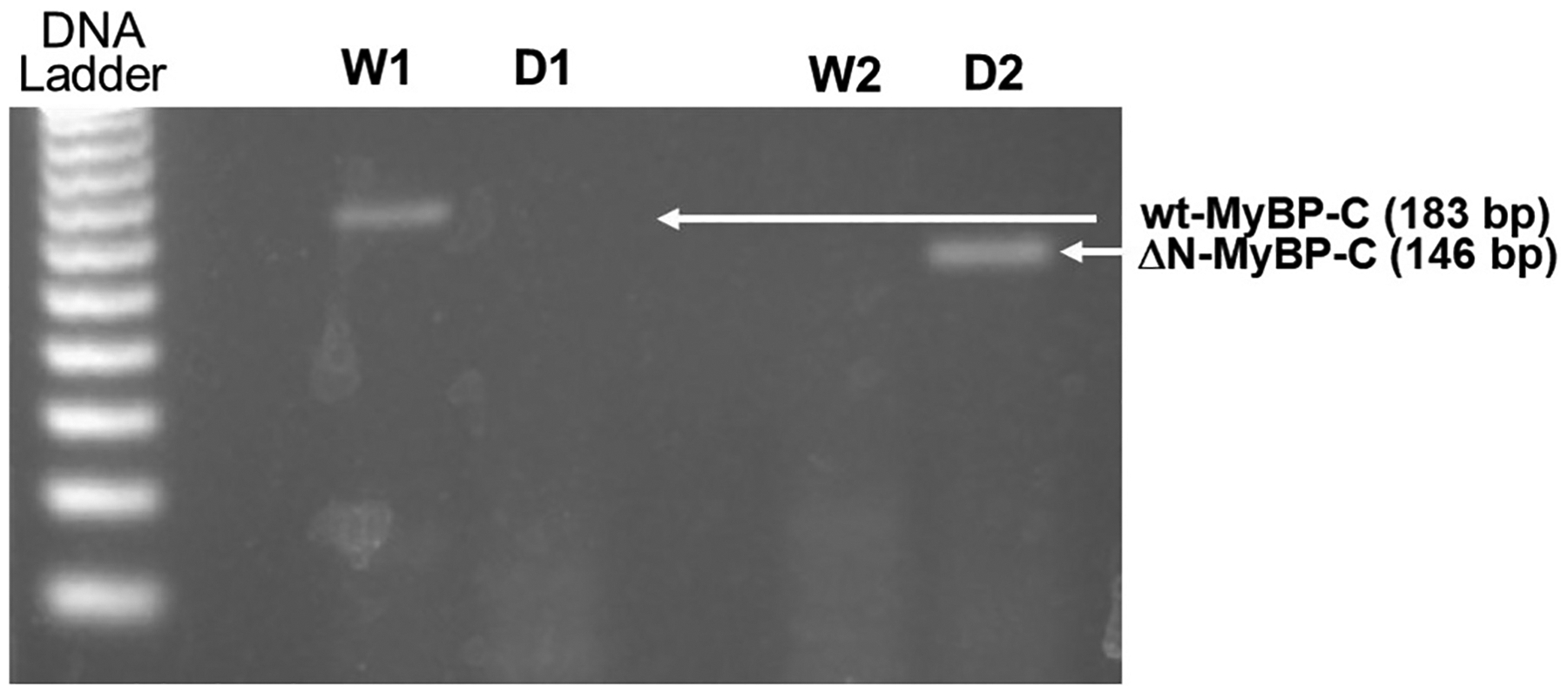
PCR results from genotyping of (A) WT rats and (B) ΔN-MYBP-C rats using two sets of primers. Each rat tissue sample was tested with both sets of primers: one set specifically recognizes and amplifies the wild-type wt-MyBP-C genome (labeled as “W” in the figure), and the other set specifically recognizes and amplifies the N-terminal region-deleted ΔN-MyBP-C genome (labeled as “D” in the figure). Lanes W1 and D1 show a band at around 183 bp only in the lane of “W1” when primer of wt-MyP-C was used, indicating the presence of WT-MyBP-C (183 bp). Lanes W2 and D2 show a band at around 146 bp only in the lane of “D2” when primer of ΔN-MyBP-C was used, indicating the presence of ΔN-MyBP-C (146 bp).

To verify the transgenic (TG) ΔN-MyBP-C rats with homozygous deletion of the C0-C2 domain of MyBP-C protein, genotyping and Western blot assays were performed. Fig. 1 shows the PCR results of the DNA extracted and amplified from our *(A)* wild-type (NTG) and *(B)* ΔN-MyBP-C (TG) rat tissue samples on 1.7 % DNA agarose gel using two sets of forward and reverse primers, one designed for detecting the wild-type (WT) MyBP-C and the other used for identifying the deletion of the C0-C2 domains (for details see the Materials and Methods section). When the deletion primers were used, we found no amplification band for our WT rats but a double band for our TG rats at 143 bp, suggesting homozygous deletion of the C0-C2 domains of the rats.

**Fig. 2.**
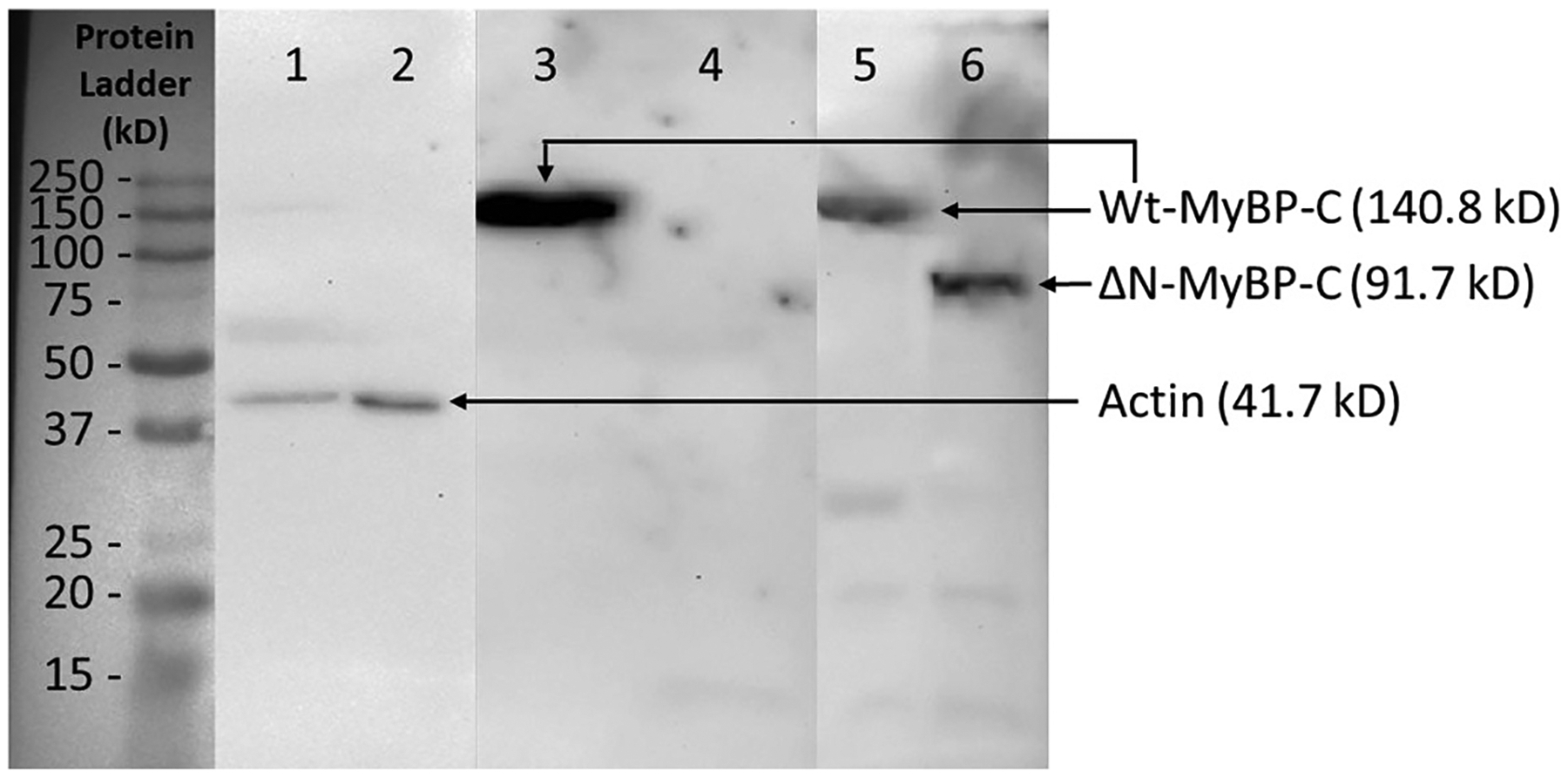
Western blot analysis of wild-type (WT) rat and ΔN-MyBP-C (TG) rat heart tissues using two different MyBP-C antibodies: one is a monoclonal antibody targeting the N-terminal region (residues 1–120) of MyBP-C (Lanes 3–4), and the other is a polyclonal antibody targeting the C-terminal region (residues 1070–1123) of MyBP-C protein (Lanes 5–6). Lanes 1, 3, and 5 are from WT rat. A clear, solid band shows at ~150 kDa in lanes 3 and 5, representing the presence of WT MyBP-C protein. Lanes 2, 4, and 6 are from ΔN-MyBP-C rat. A solid band shows in lane 6 at ~100 kDa, indicating the truncation of the C0-C2 region of MyBP-C protein. Lanes 4 show no band, indicating that the MyBP-C protein with its C0-C2 region truncated cannot be detected by the antibody targeting the N-terminal region of MyBP-C, confirming the deletion of C0-C2 from MyBP-C in the ΔN-MyBP-C rat. Actin antibody is used in lanes 1 and 2 to confirm there is comparable protein load in each lane. The WT actin band in lane 1 is not as strong as the ΔN-MyBP-C actin band in lane 2; the same case can be found in lanes 5 and 6, the WT band is not as strong as the ΔN-MyBP-C band.

Fig. 2 shows the results of the Western blot analysis of muscle fibers from both WT rats and ΔN-MyBP-C rats with 2 primary antibodies, targeting the C-terminal and N-terminal regions of MyBP-C, respectively (for details see the Materials and Methods section). Both antibodies identified a single protein band of MyBP-C at the molecular weight of 144 kDa, shown in lanes 1 and 2 for WT rats. Lanes 3 and 4 show no band for TG rats targeted with the N-terminal antibody, indicating the N-terminal region of the TG rats was deleted. Lanes 5 and 6 show the band (~91 kDa) of N-terminal-deleted MyBP-C extracted from TG rats targeted with C-terminal antibody; this indicates the TG rats had the N-terminal-truncated MyBP-C with the correct molecular size. These results provide evidence of the deletion of the C0-C2 domains of MyBP-C in our ΔN-MyBP-C rats.

### 2.2. SL effects of the N-terminus of MyBP-C on force development of skinned myocardial fibers

**Fig. 3.**
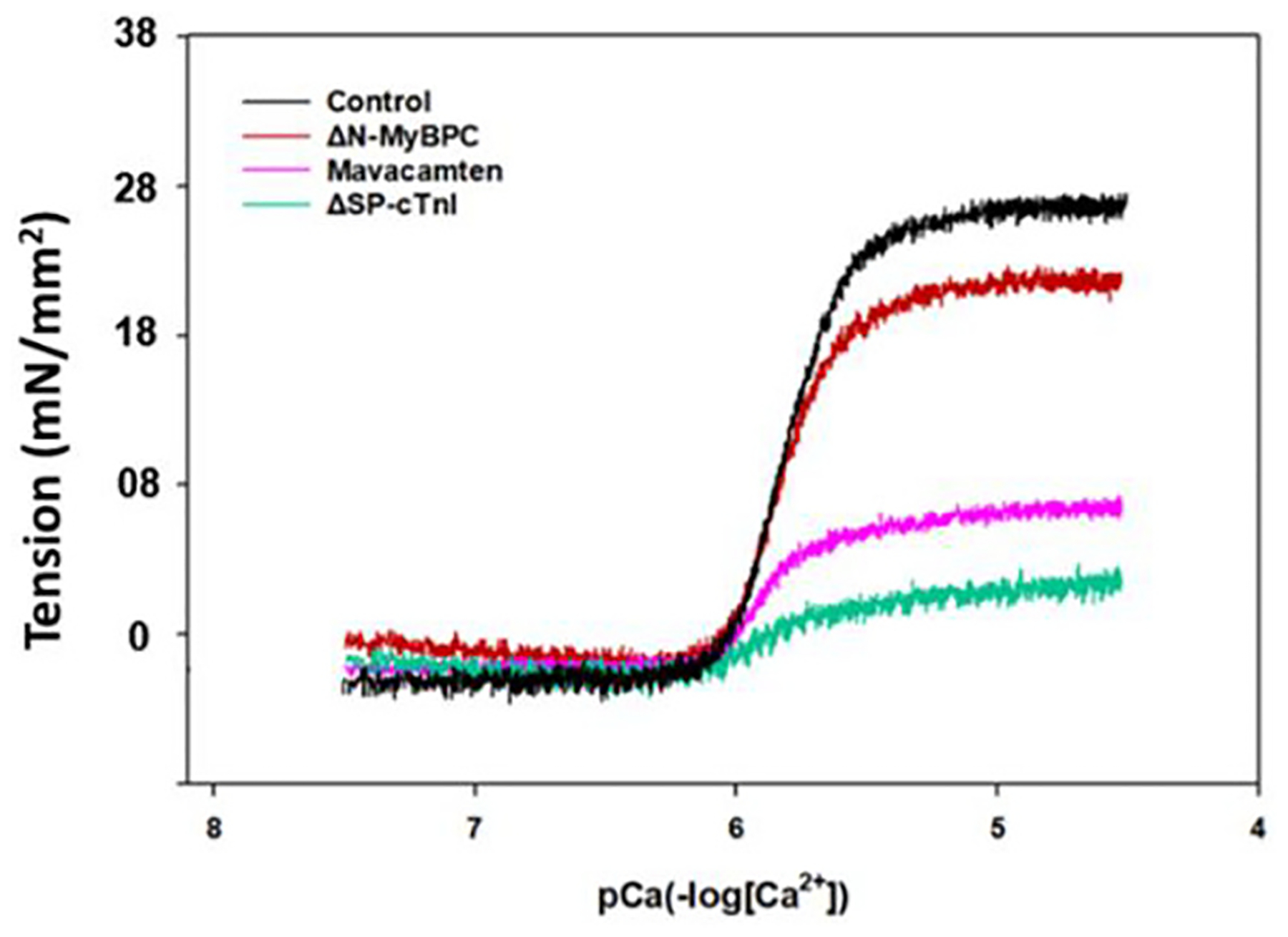
Force tension of skinned WT fibers, skinned WT fibers plus 5 μM mavacamten, skinned WT fibers reconstituted with troponin containing ΔSP-cTnI, and skinned ΔN-MyBP-C fibers at SL 2.2 μm. The curves shown here represent compiled data from multiple measurements, and the errors calculated from STD are shown in Table 2.

To examine the potential effects of the C0-C2 domains of MyBP-C on the SL–force relationship of the myocardium, Ca^2+^-activated force measurements were performed on the skinned myocardial fibers obtained from the hearts of both WT and ΔN-MyBP-C rats. The average maximum active force of skinned myocardial fibers from WT rats was 26 ± 0.8 mN/mm^2^ at SL 1.8 μm and 28 ± 0.9 mN/mm^2^ at SL 2.2 μm, whereas the average maximum active force of skinned fibers from ΔN-MyBP-C rats was 21 ± 0.7 mN/mm^2^ at SL 1.8 μm and 22.5 ± 0.8 mN/mm^2^ at SL 2.2 μm (Fig. 3). Deletion of C0-C2 domains of MyBP-C protein attenuated the maximum myofilament active force by 21 ± 4 % at short SL and by 18 ± 3 % at long SL, suggesting that the N-terminus of the MyBP-C plays an important role in modulating the active force development of myocardium at both short and long SLs. An important consideration is the effect of the lack of the C0-C2 regions of MyBP-C on diastolic cross-bridge states. Current theories have designated that an interacting head motif (IHM) of diastolic myosin heads is responsible for either a disordered relaxed (DRX) or ON state of myosin heads, which are responsible for force development with activation of the myofilaments or in a super relaxed or OFF state (SRX) [41,42]. In this manuscript we have employed the terms SRX and DRX that are common in the literature with full recognition that this concept may be more related to biochemical states than structural XB states [43,44]. A documented role of the N-terminal domains of MyBP-C is to confine myosin heads in the (SRX) state and reduce the population of DRX myosin heads [45]. Thus, it is expected that more force should be developed once the C0-C2 is removed because more myosin heads will have shifted to the DRX pool.

**Fig. 4.**
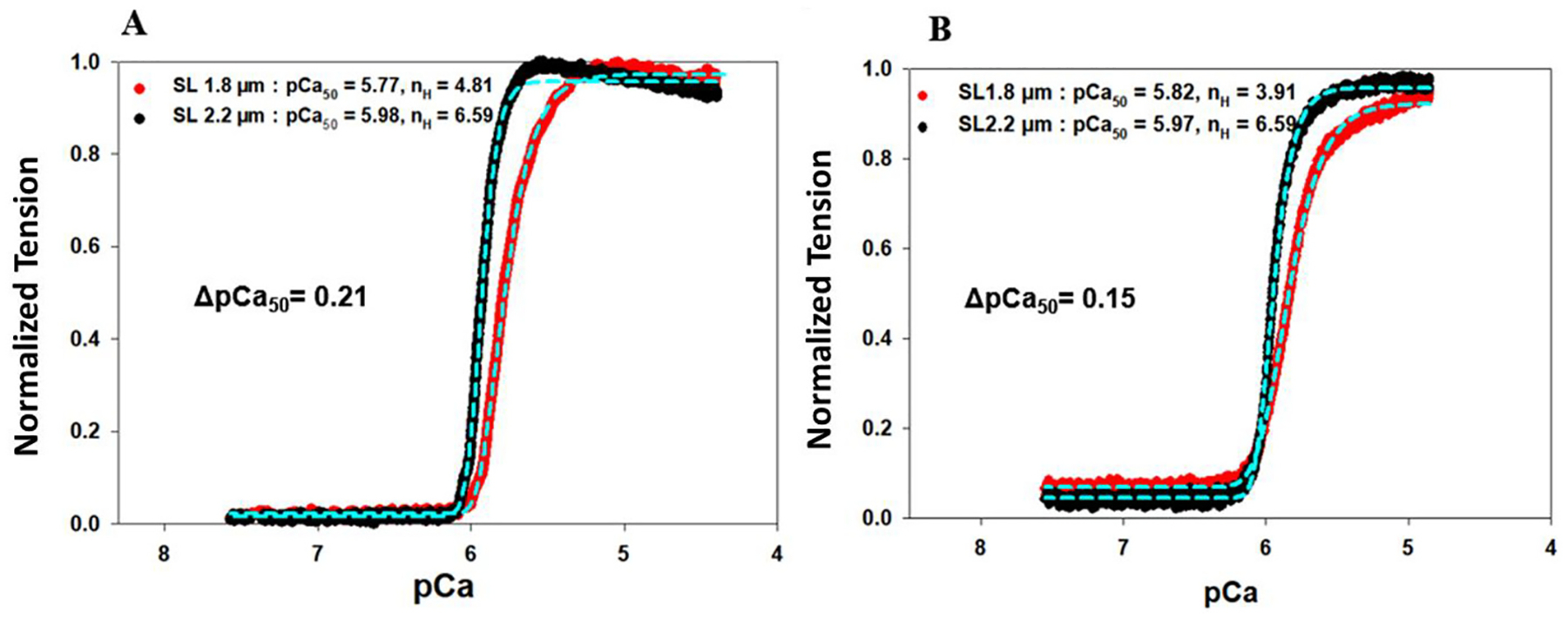
Normalized changes in tension development *vs*. Ca^2+^ titration of skinned myocardial fibers from (A) WT rats and (B) ΔN-MyBP-C rats reconstituted with a troponin complex containing cTnC (13C/51C)^AEDANS-DDPM^, WT-cTnT, and cMyc-cTnI. The measurements were performed at SL 1.8 (red) and 2.2 μm (black). Data (solid lines) were fitted to a 4-parameter Hill equation (cyan dashed lines) to derive the Ca^2+^ sensitivity (pCa^50^) and slope n^H^ (the Hill coefficient) at various SLs, which are given in the legend. The curves shown in both A and B represent compiled data from multiple measurements (N_fiber_ = 6 for A, and N_fiber_ = 5 for B), and the errors calculated from STD and significance analysis are shown in Fig. 8 and Table 2.)

We also measured the tension development of the skinned fibers *vs*. Ca^2+^ titration at various sarcomere lengths to gain information on how C0-C2 deletion affects SL-dependent activation. Fig. 4 shows the normalized force developments of *(A)* WT and *(B)* ΔN-MyBP-C fibers *vs*. Ca^2+^ titration from pCa 8.3 to pCa 4.3 at SL 1.8 μm *(red)* and SL 2.2 μm *(black)*, respectively. The calcium sensitivity, pCa_50_, of WT myocardial tension development is 5.77 ± 0.01 at SL 1.8 μm and 5.98 ± 0.02 at SL 2.2 μm (Fig. 4A). The SL-induced change in pCa_50_ (ΔpCa_50_) is 0.21. However, in the absence of C0-C2 domains, the ΔpCa_50_ reduced to 0.15, a decrease of 28.6 %. Data analysis suggests the observed decrease in force-ΔpCa_50_ induced by C0-C2 deletion is caused by two factors: One is the C0-C2 deletion increasing the Ca^2+^ sensitivity of the active force development of myocardial fibers at short SLs by 0.05 units, from a pCa_50_ of 5.77 ± 0.01 to 5.82 ± 0.01, and the other is the Ca^2+^ sensitivity of the active force of the fibers remaining unchanged at the long SLs. The SL-induced changes in force tension and Ca^2+^ sensitivity obtained from analyzing these titration curves are summarized in Table 1. These observations suggest that the C0-C2 domains prefer interacting with myosin at short SLs, which are known to constrain myosin heads in a super-relaxed (SRX) [46]; removal of the C0-C2 domains will allow the myosin heads to shift from the SRX state to a disordered relaxed (DRX) or weakly bound state.

### 2.3. Effects of the C0-C2 domains on SL-induced change in Ca^2+^ sensitivity of troponin in the presence/absence of strong XBs

To understand further the functional role of the N-terminal region of MyBP-C in the LDA of myocardial contraction, we investigated how the N-terminus of MyBP-C affects the Ca^2+^ sensitivity of troponin regulation in response to strong myosin–actin XBs. The Ca^2+^ sensitivity of troponin within skinned myocardial fibers can be examined by monitoring changes in the FRET fluorescence intensity of cTnC(13C/51C)_AEDANS-DDPM_ reconstituted skinned myocardial fibers under various conditions. Fluorophores AEDANSE and DDPM attached to residues 13 and 51 within the N-domain of cTnC serve as FRET donors and acceptors, respectively. When cTnC(13C/51C)_AEDANS-DDPM_ is reconstituted into skinned myocardial fibers, changes in the fluorescence intensity of FRET between the donor and acceptor can be used to monitor the Ca^2+^ sensitivity of troponin structural change within the myofilament structure in response to Ca^2+^ binding to the N-domain of cTnC. This approach was used in our previous studies to examine changes in the Ca^2+^ sensitivity of thin filament in regulating skinned myocardial functions under various conditions ([47] and the 1st manuscript).

In this experiment, we prepared two groups of skinned myocardial fibers from ΔN-MyBP-C rats: *group I* fibers—reconstituted with the troponin complex containing cTnC(13C/51C)_AEDANS-DDPM_, WT-cTnT and cMyc-cTnI (WT) and *group II* fibers—reconstituted with troponin complex containing cTnC(13C/51C)_AEDANS-DDPM_, WT-cTnT, and ΔSP-cTnI in which the switch peptide of cTnI was replaced by a flexible (GGGS)_3_ linker. The Ca^2+^-dependent structural opening of the cTnC N-domain, which is equivalent to the Ca^2+^ sensitivity of the troponin/thin filament of myocardial fibers, was determined by monitoring changes in the fluorescence intensity of FRET between the donor (AEDANS) and the acceptor (DDPM) of cTnC(13C/51C)_AEDANS-DDPM_ in response to Ca^2+^ titration under various SL conditions.

**Fig. 5.**
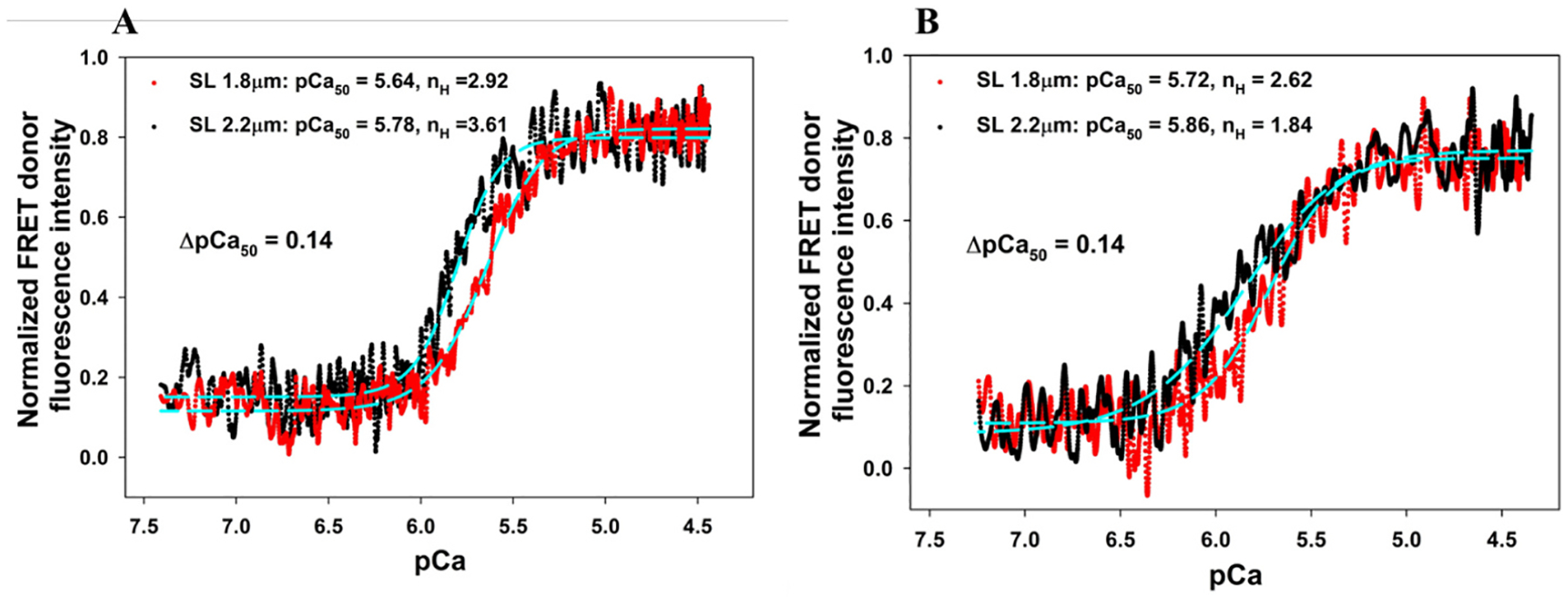
Normalized changes in FRET fluorescence intensity *vs*. Ca^2+^ titration of skinned myocardial fibers from ΔN-MyBP-C rats (A) reconstituted with Group I troponin complex containing cTnC(13C/51C)_AEDANS-DDPM_, WT-cTnT, and cMyc-cTnI (WT) and (B) reconstituted with Group II troponin complex containing cTnC (13C/51C)_AEDANS-DDPM_, WT-cTnT, and ΔSP-cTnI at SL 1.8 μm (red) and SL 2.2 μm (black), respectively. The data (dotted lines) were fitted to a 4-parameter Hill equation (cyan dashed lines) to extract the Ca^2+^ sensitivity (pCa_50_) and slope n_H_ (Hill coefficient) of the force–pCa relationship values at 1.8 and 2.2 μm SL, which are shown in the legend. The curves shown in both A and B represent compiled data from multiple measurements (N_fiber_ = 5 for A, and N_fiber_ = 6 for B), and the errors calculated from STD and significance analysis are shown in Fig. 8 and Table 2.)

Fig. 5 shows Ca^2+^ titrations *vs*. the normalized changes in the FRET donor fluorescence intensity of the skinned myocardial fibers (A) reconstituted with the group I troponin complex containing WT cMyc-cTnI and (B) reconstituted with the group II troponin complex containing ΔSP-cTnI. The Ca^2+^ titrations were performed from pCa 8.3 to pCa 4.3 at SLs of 1.8 μm *(red)* and 2.2 μm *(black)* for both group I and group II. Each of the titration curves was fitted to the Hill equation to derive pCa_50_ and the Hill coefficients (*n*_*H*_) as described previously [48,49], and the obtained results are summarized in Table 1.

For the group I fibers (Fig. 5A), in which the presence of cMyc-cTnI allowed active myosin–actin XB interactions, pCa_50_ derived from FRET-Ca^2+^ titrations were 5.64 ± 0.03 at 1.8-μm SL and shifted to 5.78 ± 0.03 at 2.2-μm SL, giving a change of 0.14 in ΔpCa_50_. These values of ΔpCa_50_ are almost the same as the 0.15 of ΔpCa_50_ obtained through force *vs*. Ca^2+^ titration (Fig. 4B and Table 1), suggesting force and FRET Ca^2+^ titrations report the same Ca^2+^-induced molecular events. Comparing these ΔpCa_50_ changes to the results obtained from myocardial fibers of WT rats (Table 1 of the 1st manuscript), deletion of the C0-C2 region of MyBP-C significantly decreases the SL-induced Ca^2+^ change in sensitivity (ΔpCa_50_), from 0.21 to 0.14. Furthermore, the deletion of C0-C2 also impacted more of the Hill coefficients obtained from FRET measurements (Fig. 5A) than that obtained from force-Ca^2+^ titration (Fig. 4B). Data obtained from our force-Ca^2+^ titrations reflect the overall cooperativity of the whole contractile apparatus including thick and thin filament components and all other regulatory processes, whereas the data obtained from our FRET-Ca^2+^ titrations reports the cooperativity of local conformational changes of troponin and feedback effects from local surroundings.

For the group II fibers, when intrinsic cTnI of the reconstituted fibers was replaced by ΔSP-cTnI, the active force development was significantly abolished (Fig. 3) because the XB inhibition imposed by ΔSP-cTnI cannot be released by Ca^2+^ binding to cTnC. This design functionally separates troponin/thin filament regulation from the component of thick filament force development. Although the force–Ca^2+^ sensitivity of this system cannot be obtained, the sensitivity of troponin to Ca^2+^ regulation can be obtained using a FRET-Ca^2+^ titration approach. Fig. 5B shows the normalized changes in the FRET fluorescence intensity of *group II* fibers *vs*. Ca^2+^ titrations from pCa 8.3 to pCa 4.3 at SLs of 1.8 μm *(red)* and 2.2 μm *(black)*. These results show that pCa_50_ derived from FRET-Ca^2+^ titrations were 5.72 ± 0.05 at short SL and 5.86 ± 0.04 at long SL, respectively. The sarcomere stretches induced a 0.14 change in ΔpCa_50_, which is the same as the ΔpCa_50_ obtained from the group I fibers (Fig. 5A), suggesting that SL-induced changes in ΔpCa_50_ are independent of the presence of myosin–actin XB interactions. It is to be noted that the Ca^2+^ sensitivities (pCa_50_) of the group II fibers obtained at both short and long SLs (Fig. 4B) were more than those of the group I fibers (Fig. 5A). These changes in Ca^2+^ sensitivities could be caused by the presence of extrinsic SP in the *group II* fibers, which could synergize the Ca^2+^-induced opening of N-cTnC. In addition, compared to the results from *group I*, the cooperativity of Ca^2+^-induced myofilament activation in *group II* samples was decreased, as suggested by the changes in the Hill coefficient (*n*_*H*_), from 2.92 to 2.28 at short SL and from 3.61 to 2.83 at long SL (Table 1). The observed decreases in cooperativity likely result from the disengagement between thin filament and thick filament caused by ΔSP-cTnI. The results from titration analysis are given in Table 1.

The N-terminal region of MyBP-C is known to interact with myosin to restrain the XB numbers and render myosin heads in the SRX state [46]; removal of the C0-C2 will release myosin heads from SRX and increase the DRX pool. Since our previous study showed that active myosin heads are required for the SL-induced myosin–troponin interaction (the 1st manuscript), removal of the C0-C2 should enhance the SL-induced ΔpCa_50_. However, our current results show deletion of the C0-C2 induced about a ~ 32 ± 3 % reduction of the SL-induced Ca^2+^ sensitivity. These results suggest that the C0-C2 domains participate in the LDA through direct interaction with actin/Tm/troponin or modulate the potential myosin-troponin interaction by interacting with myosin heads within the C-zone of the sarcomere. To understand further the nature of the observed effects of C0-C2 deletion on SL-induced changes in Ca^2+^ sensitivity, we performed the following experiments in which we perturbed the activation state of myosin heads using the drug mavacamten.

### 2.4. Synergic effects of myosin heads and the C0-C2 domains of MyBP-C on SL-induced changes in the Ca^2+^ sensitivity of skinned myocardial fibers

**Fig. 6.**
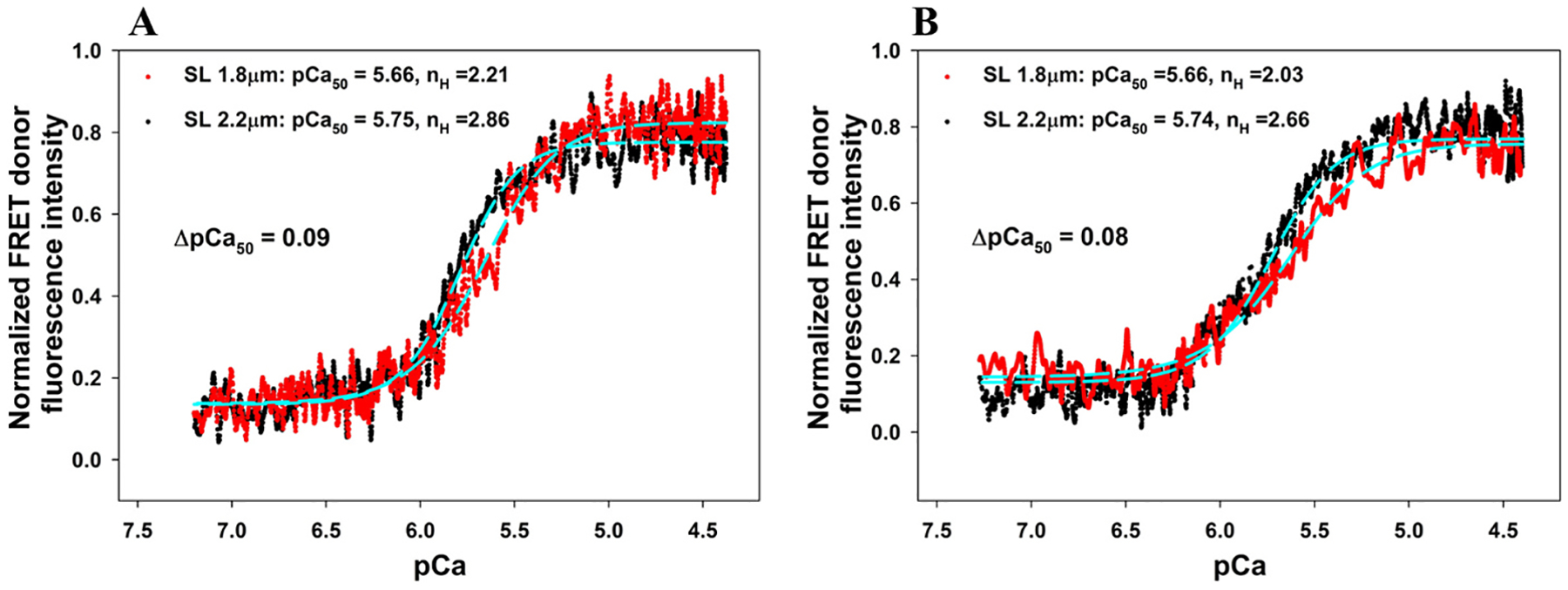
Ca^2+^ titration *vs*. the normalized changes in FRET donor fluorescence intensity of ΔN-MyBP-C fibers reconstituted with (A) Group I troponin complex containing cTnC(13C/51C)_AEDANS-DDPM_, WT-cTnT, and cMyc-cTnI (WT) and Group II troponin complex containing cTnC(13C/51C)_AEDANS-DDPM_, WT-cTnT, and ΔSP-cTnI (B) at SL 1.8 μm (red) and SL 2.2 μm (black), respectively, in the presence of 5 μM mavacamten. The data (dotted lines) in the figures were fitted to a 4-parameter Hill equation (cyan dashed lines) to extract the Ca^2+^ sensitivity (pCa_50_) and slope n_H_ (Hill coefficient) of the force–pCa relationship values at 1.8 and 2.2 μm SL; these are listed in the legends. The curves shown in both A and B represent compiled data from multiple measurements (N_fiber_ = 8 for A, and N_fiber_ = 9 for B), and the errors calculated from STD and significance analysis are shown in Fig. 8 and Table 2.)

Fig. 6 shows the results of FRET-Ca^2+^ titrations of both *(A) group I* and *(B) group II* reconstituted myocardial fibers from ΔN-MyBP-C rats in the presence of 5 μM mavacamten at SL 1.8 μm *(red)* and SL 2.2 μm *(black)*. The values of pCa_50_ and the Hill coefficients (*n*_*H*_) derived from the analysis of each titration are given in Table 1. For *group I* fibers, which were reconstituted with the troponin complex containing cMyc-cTnI (WT), the presence of the drug reduced SL-induced changes in ΔpCa_50_ from 0.14 (Fig. 5A) to 0.09. For *group II* fibers, in which the presence of ΔSP-cTnI inhibits XB formation, the presence of the drug desensitized troponin to Ca^2+^ at both the long SL and the short SL (Table 1), which led to a decrease in ΔpCa_50_ from 0.14 (Fig. 5B) to 0.08. For both *group I* and *group II* samples, the cooperativity of troponin to Ca^2+^ binding was not significantly perturbed by the presence of mavacamten (Table 1).

It is known that mavacamten increases and stabilizes the SRX population of cardiac myosin and reduces population of DRX state [50]; 5 μM mavacamten was used in this experiment to manipulate the populations of the DRX/SRX states of the myosin heads of the skinned myocardial fibers. The maximum Ca^2+^-activation force of the myocardial fibers of ΔN-MyBP-C from *group I* was reduced by 60 ± 3 % in the presence of 5 μM mavacamten (Fig. 3), which is consistent with the role of the drug in increasing the SRX population of the myosin heads and thus reducing the myocardial contractility. In contrast, the presence of the drug only blunted ΔpCa_50_ by about one-third. This change may reflect a significant portion of the myosin still being in the weakly bound (DRX) state in the presence of 5 μM mavacamten. This was investigated in the next experiment using an *in vitro* stopped-flow technique.

### 2.5. SRX/DRX population of myosin within skinned myofibrils under various conditions

**Fig. 7.**
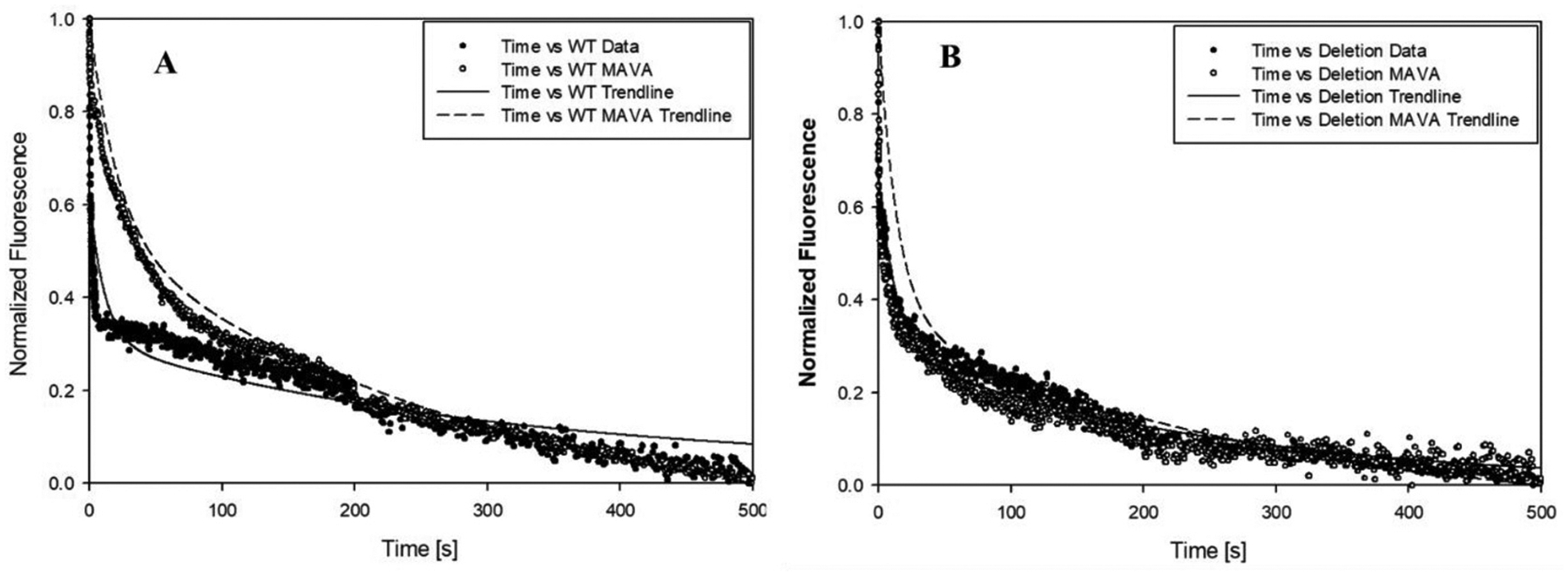
Typical s*topped-flow fluorescence decays* of the myosin-bound mant-ATPs after their replacement by natural ATP molecules at 20 °C. A: kinetic decay of myofibrils from WT rat heart in the absence and presence of 5 μM mavacamten. B: kinetic decay of myofibrils from ΔN-MyBP-C rat heart in the absence and presence of 5 μM mavacamten. Each decay curve was reconstructed from three time-scale data sets obtained at 25 s, 200 s and 500 s, respectively, and each data set contains 1000 data points. Each reconstructed decay (dotted line) was fitted to a double-exponential function (solid line) to derive decay rates and associated amplitudes.

To understand further the relation of SRX/DRX populations of myosin heads with the C0-C2 truncation and the presence of mavacamten, we used a stopped-flow (SF) technique to examine changes in the SRX and DRX populations in our myocardial tissues. Briefly, skinned myofibrils from either WT rats or ΔN-MyBP-C rats incubated with mant-ATP were rapidly mixed with unmodified ATP using our stopped-flow device in the absence/presence of 5 μM of mavacamten (see Materials and Methods section). Fig. 7 shows a set of typical SF decays: fluorescence of the myosin-bound mant-ATPs decays after their binding replacement by natural ATP molecules. The decay can be fitted to a double-exponential function, yielding a fast-decay component with a decay time in the range of 0.01–0.09 s^−1^ and a slow-decay component with a decay time of 0.001–0.009 s^−1^ [51]. The fast-decay component is associated with myosin molecules in the DRX state, while the long-decay component is associated with myosin heads in the SRX state [51–54]. The amplitude associated with each decay component represents the population of that group of myosin heads. The results of the population analysis of each myosin group are summarized in Table 2.

For the skinned myofibrils from WT rats in the absence of Ca^2+^, the SRX/DRX population ratio was 46.6/53.1; as expected, the presence of Ca^2+^ shifted the population ratio to 16.6/83.4; *i.e*., more myosin heads moved to the weakly bound state favoring myosin–actin interaction. When the mavacamten was added to the relaxed myofibrils, the population ratio changed to 61.5/38.5; *i.e*., more myosin heads were in the SRX state. These results are consistent with previous reports in literature [51,55]. For the skinned myofibrils from ΔN-MyBP-C rats, in which the C0-C2 was truncated, the SRX/DRX ratio was decreased to 40.6/59.4 in the relaxed state, suggesting that decreased numbers of the myosin heads in the SRX state when the C0-C2 of MyBP-C was removed. This observation is consistent with the role of the C0-C2 of putting myosin heads in SRX [56]. Interestingly, adding 5 μM of mavacamten to the same myofibril sample induced insignificant changes in the population ratio, implying that mavacamten may be partially or fully reliant on full length cMyBP-C to function properly, as previously study suggested [56].

## 3. Discussion

Sarcomere stretch-induced increases in the Ca^2+^ sensitivity of myofilaments is a hallmark of LDA. In our previous study we reported that the SL-induced increase in troponin-Ca^2+^ sensitivity in skinned rat myocardial fibers is independent of the myosin–actin XB interaction; we attributed this SL-induced effect to an interaction between weakly bound myosin heads and troponin possibly caused by a reduced sarcomere interfilament spacing or increased titin-induced strain on the thick filament. This study reports our continuous efforts to understand the mechanism underlying the observed XB-independent SL-induced changes in troponin Ca^2+^ sensitivity. Using our ΔN-MyBP-C transgenic rat model in which the C0-C2 domains of MyBP-C were deleted and a previously established *in situ* mechanical/FRET-based skinned myocardial platform in which the myosin–actin XB can be effectively inhibited using ΔSP-cTnI mutant to replace intrinsic wt-cTnI (the 1st manuscript), we were able to investigate the potential synergetic role of C0-C2 of MyBP-C and the state of myosin heads in the observed XB-independent SL-induced increase in ΔpCa_50_ changes of the myofilament of skinned myocardium. Two primary findings were observed in this study.

**Fig. 8.**
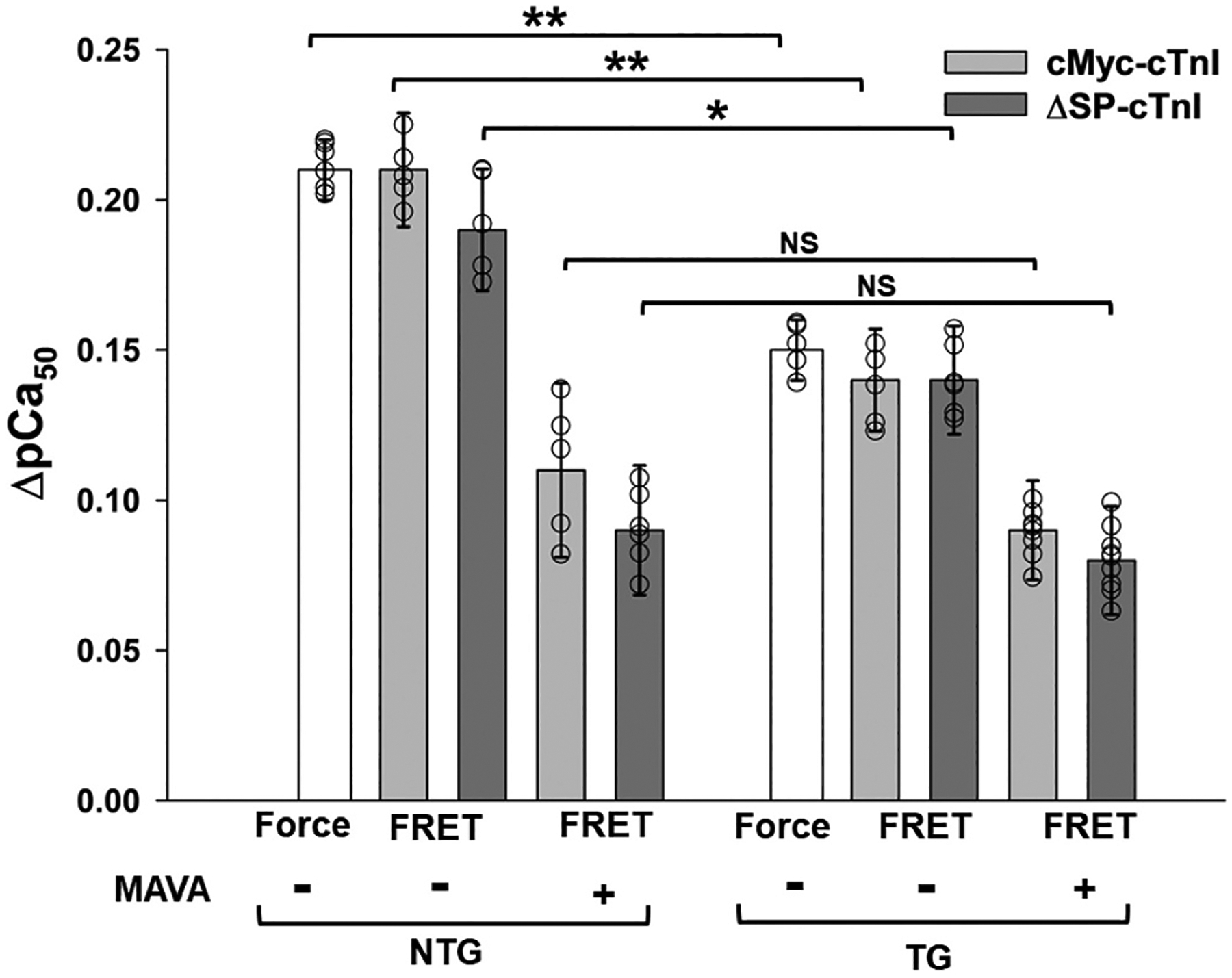
The bar chart of summarizing force-ΔpCa_50_ and FRET-ΔpCa_50_ of wild-type (labeled as “NTG”) and transgenic ΔN-MyBP-C (labeled as “TG”) rat cardiac muscle fibers reconstitution with the troponin complex containing (1) WT-cTnT, cMyc-cTnI (WT), and cTnC(13C/51C)_AEDANS-DDPN_ (grey filled bar), and (2) WT-cTnT, ΔSP-cTnI, and cTnC(13C/51C)_AEDANS-DDPN_ (diagonal stripe bar), with 5 μM mavacamten (labeled as “+” or without mavacamten (labeled as “−”). Values are reported as mean ± SD. **, *p* < 0.01, *, *P* < 0.05, NS, no significant changes.

Firstly, removal of the C0-C2 domains reduced the SL-induced changes in ΔpCa_50_ by ~25 % regardless of the presence/absence of strong XBs (Fig. 8). This finding reconfirms that strong myosin–actin cross-bridge interactions are not involved in transducing SL mechanic effect to troponin-Ca^2+^ sensitivity. Furthermore, considering the location of MyBP-C in sarcomere, this finding suggests that the presence of the C0-C2 domains may enhance SL-induced Ca^2+^ sensitivity of the thin filament in the C-zone compared to other zones of sarcomere. This asymmetric distribution of the SL-induced Ca^2+^ sensitivity across the sarcomere may give an advantage that counterbalances the nonuniform activation of myocardium caused by the spatial and temporal gradient in free Ca^2+^ across the sarcomere [57], which further suggests the critical functional importance of the N-terminal domains of MyBP-C in LDA.

Secondly, the observed SL stretch-induced elevation in troponin Ca^2+^ sensitivity depended on the population of myosin heads in the weakly bound (DRX) state (Table 1). For example, when 5 μM mavacamten was applied to the skinned myocardial fibers to reduce the population of myosin heads in DRX state and increase the population of myosin heads in SRX state (Table 2 and [51]), the observed SL-induced changes in ΔpCa_50_ reduced from 0.20 to 0.21 to 0.09–0.11 regardless of the presence/absence of the C0-C2 domains and the strong XBs (Fig. 8), suggesting the critical role of myosin heads in the RDX state in the mechanism of the observed SL-induced changes in ΔpCa_50_. Furthermore, the observed ~25 % reduction in the SL-induced changes in ΔpCa_50_ caused by C0-C2 domain deletion was not additive to the reduction caused by mavacamten-induced reduction of the myosin heads in DRX state. Although these results may shine a light on the mechanism underlying the role of the C0-C2 domains in the observed SL-induced Ca^2+^ sensitivity changes of myofilament regulation, it should be caution to our interpretation on these results. As mentioned above, our ΔN-MyBP-C transgenic rat model is not fully characterized; deletion of C0-C2 can potentially have secondary effects on mechanics ro the ratio of DRX/SRX of myocardial fibers, which may impact our results reported here. However, these potential effects are not investigated in this study.

One of the proposed mechanisms to account the role of MyBP-C in myofilament Ca^2+^ regulation is potential direct interactions between the C0-C2 domains and actin filament, including troponin complex. Evidence supporting this mechanism includes several *in vitro* and *in situ* studies. For example, a report by Risi et al. [58] suggests that the first two domains (*i.e*. C0 and C1) of human MyPB-C cooperate to activate the thin filament through interaction between C1 and tropomyosin with C0 as a stabilizer for the interaction. Early work by Palmer et al. [59] suggests that the presence and phosphorylation of the N-terminus of MyBP-C provides structural support and radial rigidity to the myofilament lattice, which may potentially affect myosin force development and myofilament regulation. More recently, a study from Rahmanseresht et al. [18] provides evidence suggesting, instead of lying along the surface of the myosin filament, the N-terminus of MyBP-C extends toward actin filaments in intact cardiac muscle, which may help initiate muscle contraction. Although these findings shine light on the direct interactions between the N-domains of MyBP-C and thin filament, it is likely that the C0-C2 deletion induced reduction in ΔpCa_50_ observed in this study may not involve in the direct interactions between the C0-C2 domains and troponin/thin filament. This is because the observed of the none-additive reductions in the SL effect caused by C0-C2 deletion and the effect caused by mavacamten treatment (Fig. 8), *i.e*., the SL-induced change in pCa_50_ (ΔpCa_50_) can be reduced to the same level by mavacamten to manipulate the status of myosin heads regardless the presence or the absence of the C0-C2 domains. Therefore, an alternative mechanism may involve the C0-C2 region of MyBP-C working together with myosin heads within the C-zone to modulate the SL effect.

It has been suggested that myosin heads are not uniformly distributed along the thick filament. The population of SRX myosin in the P-zone and D-zone of the thick filament, where no MyBP-C exists, is much smaller than the population of SRX myosin in the C-zone, where MyBP-C is located [60]. Thus, myosin heads in different zones may contribute to the SL-induced increase in ΔpCa_50_ differently. For example, in the D-zone the contribution may be primarily associated with SL-induced interactions between troponin and DRX myosin heads, while in the C-zone the N-terminal domains of MyBP-C may be involved in SL-induced myosin-troponin interactions that enhance the SL-induced effect on Ca^2+^-troponin sensitivity. Recent high-resolution structural studies of cardiac myosin filament [28,29] revealed that there are three distinct types of crown structures in myosin heads (crowns 1, 2 and 3), arranged in 43-nm triplets, and that each type of crown interacts with MyBP-C and titin differently depending on its position. Among these crowns, crown 2, a possibly disordered relaxed (DRX) myosin head (also called CrD) is unique. The tails of the CrD from three consecutive 43-nm repeats form a specific coalescence, a sheet-like docking platform, to accommodate bindings of the C6-C10 domains of MyBP-C, whereas the head of each CrD can potentially interact with the C0-C2 domains of MyBP-C [29], thus modulating myosin activity through the interaction with CrD myosin heads, with fine-tuning through phosphorylation of the M-domain of MYBP-C. In our study, the presence of the C0-C2 of MyBP-C proteins, which are located in the C-zone, contributed ~25 % of overall SL-induced effects on ΔpCa_50_ (Fig. 8). This effect is likely caused by this mechanism. Under this mechanism, the disordered DRX myosin head is likely the major player in the observed SL-induced myosin–troponin interaction on ΔpCa_50_, while the C0-C2 domains of MyBP-C likely play a modulatory role in the proposed SL-induced myosin–troponin interaction. Our results are consistent with this mechanism. Although our results provide new insight into the potential role of the N-terminal domains of MyBP-C in length-dependent myofilament activation, how phosphorylation affects the observed role cannot be determined from this study since our measurements were performed without controlling the phosphorylation status of the muscle fibers.

### 3.1. A tentative model for sarcomere length-dependent activation

Based on the results obtained from this study, we update the model that was proposed in our previous publication (the 1st manuscript) to account for the roles of myosin–actin interaction, myosin–troponin interaction, and the N-domains of MyBP-C in sarcomere stretch-induced activation of myofilament. Briefly, this model describes SL stretch-induced changes in the states of myosin heads and their interactions with actin and troponin in outside C-zone region, as well as the role of the N-domain of MyBP-C in modulating these changes in the C-zone. In this model, myosin heads are in either the SRX state or the RDX state, and transition between these two states is SL-dependent and can be modulated by the N-domains of MyBP-C in the C-zone. For the region outside of the C-zone of a sarcomere, there are a total of 9 myosin double heads within a 43-nm length of thick filament. As discussed earlier, cryo-EM studies revealed that these 9 myosin double heads form three populations: CrH (Cr1), CrD (Cr2) and CrT (Cr3), with distinct structural arrangement within the myosin filament [28,29]. Among these populations, the heads of CrD are unique. They exhibit much wider radial distance with their free heads projected further outwards, making them closer to the thin filament. Therefore, the myosin heads of CrD could participate in Ca^2+^ sensitivity regulation through SL-induced interaction with troponin in the actin/Tm region interacting with cTnT-T1 and residues 182–229 of cTnT-T2 (the 1st manuscript). The myosin heads of CrH and CrT in the RDX state are ready for participating in SL-induced force development by binding to actin filament. Within the C-zone, there are 3 MyBP-C molecules and 9 myosin double heads with 3 unique populations (CrH, CrD and CrT) within each 43-nm length of thick filament. Again, the unique structural locations of the myosin molecules of CrD allow their tail regions to interact with the C6-C10 domains of MyBP-C, whereas the heads of CrD can interact with the C0-C2 domains of MyBP-C. Therefore, it is possible that the N-domains of MyBP-C modulate the SL-induced interaction between the myosin heads of CrD and troponin with fine-tuning through phosphorylation of the M domain of MyBP-C.

*How does the model work?* As sarcomere is stretched under diastole (Graph 1), it increases titin-based passive tension by straightening spring-like domains, or kinks [29]. The stretching of titin strands can have multiple structural and functional impacts on myofilament. Firstly, because of interactions between titin strands and tails of myosin molecules, the mechanical stretching causes myosin tails to slide past each other and promotes the shifting of myosin from SRX to DRX [61,62], thus increasing the population of weakly bound DRX myosin heads. In the C-zone, the shift from SRX to DRX is likely caused by disruption of the interactions between myosin heavy chains and MyBP-C [28,29]. Secondly, sarcomere stretch-induced decrease in interfilament spacing [63] will allow the mobile heads of CrD myosin in the RDX state to overreach the troponin, leading to potential interactions in the actin/Tm thin filament region containing cTnT-T1 and residues 182–229 of cTnT-T2. The interactions will then propagate to the N-domain of cTnC *via* the IT arm and the N-terminus of cTnI, thus sensitizing troponin to Ca^2+^ binding in the diastolic state and leading to a partial activation of the thin filament to allow myosin–actin XB formation. This provides a basis for accounting for the SL-induced enhancement of Ca^2+^ sensitivity of thin filament. At the same time, the myosin heads of CrH and CrT, after being moved from the SRX state to the DRX state by stretching of the sarcomere, will be available and ready for actin interaction at the end of the diastolic phase, thus accounting for the SL-induced enhancement of systolic force that follows. Because each of the myofilament components or proteins, such as troponin/thin filament, myosin, MyBP-C and titin, play various functional and structural roles in these hypothetical processes, our proposed model provides a platform for future study of the role of each component in SL-induced changes in Ca^2+^ sensitivity and force development and examination of how cardiomyopathy mutations of each protein alter its role in LDA. For example, many hypertrophic and dilated cardiomyopathy mutations have been identified on each of these proteins [64]. However, different cardiomyopathy mutations affect contractility of human heart muscle differently through alterations of SL-dependent changes in either Ca^2+^ sensitivity or force development or both [64]. If our model is verified, these observations can then be explained by the locations of the mutations within the LDA model, *i.e*., the functional effect of a mutation on LDA will depend on the location of the mutation on the functional protein and whether the mutation affects the pathway of SL-induced Ca^2+^ sensitivity change or the pathway of SL-induced change in tension development, or both. The model will also provide a platform for studying phosphorylation effects on each pathway and understanding the role of myofilament cooperativity in mechanical stretch-induced activation of thin filament and myosin thick filament.

## 4. Materials and methods

### 4.1. Creation of transgenic model

Homozygous Sprague Dawley ΔN-MyBP-C transgenic rats were created by working with the Animal Modeling Core facility at University of Missouri. Briefly, two sgRNAs with sequences of 5’ GTTCTCAAGATGCCTGAGCC 3′ and 5’ TCCTCCCTGGACAGAACCCC 3′ were designed to target rat Mybpc3 exons 1 and 14 using the CRISPR RGEN Tools website maintained by the Center for Genome Engineering Institute (Korea) and the CCTop website maintained by the Centre for Organismal Studies (Heidelberg) to calculate off-target scores. These sgRNAs were ordered as a chemically modified synthetic sgRNA through Synthego. The chemical modifications on the sgRNA were 2’-*O*-methyl analog and 3′ phosphorothioate internucleotide linkages at the first three 5′ and 3′ terminal RNA residues. When used with CRISPR-mediated genome editing these sgRNAs were designed to generate a 6359 bp deletion in the genomic DNA sequence, excising part of exon 1, all of exons 2–13, and part of exon 14 of rat Mybpc3.

A mixture containing a final concentration of 3.0 μM each sgRNA and 2.0 μM enhanced specificity Cas9 protein (Sigma) was made immediately prior to electroporation. CRISPR sgRNA/Cas9 RNP complexes were formed by gently mixing the sgRNAs and Cas9 protein together and incubating at room temperature for 10 min. Using a NepaGene21 electroporator along with a 1.5-mm gap glass slide electrode, Sprague Dawley rat zygotes were electroporated under the following conditions: Poring pulse: 40 V, 3.5 ms length, 50 ms interval, 10 % decay rate, positive polarity (x4 pulses) Transfer pulse: 5 V, 50 ms length, 50 ms interval, 40 % decay rate, alternating polarity (x5 pulses). Electroporated zygotes were surgically transferred to pseudo pregnant surrogate females the same day as the electroporation. NTac:SD embryo donor females (3 weeks of age), stud males (10 weeks of age) and surrogate females (8 weeks of age) were purchased from Taconic (NTac: SD). Eventually, two pairs of homozygous Sprague Dawley ΔN-MyBP-C transgenic rats (2 females, #657CH and #658CH, and 2 males, #655CH and #656CH) with truncation of the N-terminal (C0-C2) region of MyBP-C were used to bred and used in this study.

### 4.2. Animal handling protocols

All the handling of our experimental animals followed the guidelines of the Institutional Animal Care and Use Committee at Washington State University and the Office of Laboratory Animal Welfare, National Institutes of Health, Bethesda, MD. Cardiac muscle fiber preparation adhered to the established protocols and guidelines of ASAF #7212 approved by the Washington State University Institutional Animal Care and Use Committee. Both WT and transgenic rats were anesthetized with 3 % isoflurane (Vet Pharma) in 95 % O_2_ and 5 % CO_2_ inhalation at a flow rate of 2 L/min. Hearts were promptly excised and placed in ice-cold dissecting solution.

### 4.3. Protein preparation

Recombinant proteins cMyc-cTnI, ΔSP-cTnI and WT-cTnT were each subcloned into the pET-3d vector and expressed in BL21 competent cells (Cat: C600003, Invitrogen). They were purified using a CM and DEAE column following the protocol previously established in our lab (27). The recombinant mutant cTnC(T13C/N51C) with double-cysteine was generated from rat cDNA clones and subcloned into a pET-3d vector, which was then used to transform BL21(DE3) cells (Invitrogen) and induced with isopropyl β-D-1-thiogalactopyranoside (IPTG) (Sigma) for protein expression. The expressed protein was purified as previously described [11,48,65,66]. Subsequently, the FRET donor 5-((((2-iodoacetyle)amino)ethyl)amino)naphthalene-1-sulfonic acid (IAEDANS) was added to purified cTnC(T13C/N51C) protein, followed by our previously established protocol [67]. To separate the singly labeled cTnC (T13C/N51C) from unlabeled and doubly labeled proteins, the protein mixture was passed through DEAE column. The singly labeled cTnC (T13C/N51C)_AEDANS_ was carefully collected and then labeled with the FRET donor N-(4-dimethylamino-3,5-dinitrophenyl) maleimide (DDPM) at the other cysteine to generate the donor–acceptor labeled cTnC(T13C/N51C)_AEDANS-DDPM_. The labeling ratio of the donor-only sample was determined spectroscopically using ε_325_ = 6000 cm^−1^ M^−1^ for AEDANS [11].

### 4.4. Fiber skinning and reconstitution with the recombinant troponin complex

The WT and transgenic rat cardiac filament fibers were collected from left ventricular papillary bundles following a protocol established previously in our lab [11,68]. Both male and female Sprague Dawley (SD) rats under 4 months of age were used in this study. The heart was quickly removed from each rat while 3 % isoflurane (in O_2_) was applied at a rate of 2 min/L. The heart was kept in ice-cold heart relaxing buffer (HR buffer) with 0.1 % protease inhibitor (Cat: 539134, EMD Millipore Corp.) until dissection. The HR buffer is composed of 50 mM BES, 30.83 mM K-propionate, 10 mM NaN_3_, 20 mM EGTA, 6.29 mM MgCl_2_, and 6.09 mM Na_2_ATP [11,68]. Only the left ventricular papillary bundle fibers were used in this protocol. The tissue bundle was extracted from the left ventricle and then dissected into uniform fibers, each 150–200 μm in diameter and 500 μm in length in HR buffer on a cooling plate at 4 °C. The fibers were skinned by incubating them overnight at 4 °C in a skinning buffer (Skinning buffer: 1 % Triton X-100 in HR buffer, with 1% protease inhibitor cocktail) with constant shaking.

Skinned cardiac muscle fibers were incubated in cTnC extraction buffer (cTnC extraction buffer: 5 mM CDTA, 0.6 mM NaN_3_, 40 mM Tris-HCl, 0.1 % protease inhibitor cocktails, pH 8.4) at 4 °C for 2.5 h with constant shaking [11,68]. The fibers were then washed twice in HR buffer at 4 °C for 5 min each after cTnC extraction, subsequently reconstituted in the protein exchange buffer with recombinant troponin complex at 4 °C (Protein exchange buffer: 50 mM BES, 30.83 mM K-propionate, 5 mM NaN_3_, 10 mM EGTA, 6.09 mM Na-ATP, 10 mM BDM, 4 mM Ben-HCl, 1 mM DTT, 0.2 mM PMSF, 0.1 % protease inhibitor cocktails, pH to 7 by KOH). WT-cTnT and cTnC(13C/51C)_AEDENS-DDPM_ were dialyzed with either cMyc-cTnI (WT) or ΔSP-cTnI, against a reconstitution buffer containing a urea gradient from 6 M to 0 M, to form the recombinant troponin complex. The concentration ratio of cTnI, cTnT, and cTnC proteins in the reconstitution buffer was 1.2:1.2:1. Fibers were incubated in the recombinant troponin complex protein exchange buffer for two 4-h sessions to ensure the protein exchange efficiency reached 80 %.

### 4.5. Genotyping of wild-type and transgenic ΔN-MyBP-C rat heart muscle fibers

Tissue samples of wild-type Sprague Dawley rats and homozygous ΔN-MyBP-C transgenic rats for genotyping were carefully collected from the ear(s) using a tissue puncher and immediately stored at −80 °C until use. Ear tissues were first digested in digestion buffer (10 mM Tris-HCl, 50 mM KCl, 1.5 mM MgCl, 0.1 % gelatin, 0.45 % NP-40, 0.45 % Tween-20, pH 8.3) on ice for 30 min. Protease K (Cat: P8107S, NEB) was added to the digestion buffer in a 1:100 ratio freshly before use. The tissue sample was then heat-shocked for 20 min at 60 °C followed by 3 min at 100 °C. It was spun at 13,300 *g* for 10 min to collect the supernatant containing the DNA. The isolated DNA solution was stored at 4 °C until use. DNA concentration was measured using a Nanodrop machine (Nanodrop 2000c, ThermoScientific).

The primer sequences for mutant alleles are 5’ AGTGAGAACACGGGACACTGTTCAG 3′ (forward) and 5’ TCACACTCAAACTCCACCCGCTGAC 3′ (reverse) (synthesized by IDT). The primer sequences for wild type alleles are 5’ AGTGAGAACACGGGACACTGTTCAG 3′ (forward) and 5’ CCTCATCCTATGCCAGTACCTAACC 3′ (reverse) (synthesized by IDT). To amplify and test the truncation alleles, a PCR assay was performed on the tissue samples. One μL of extracted DNA solution was added to 18 μL of PCR SuperMix buffer (1.1×) (Cat: 10–572–014, Invitrogen) with 0.5 μM of each forward and reverse primer, resulting in a total volume of 20 μL. The PCR reaction was run using the following settings: 95 °C for 3 min, then 33 cycles of 95 °C for 30 s, 61 °C for 30 s and 72 °C for 1 min.

A1. DNA gel (7 %) was freshly prepared each time before use by adding 0.85 g of agarose to 50 mL of 1× TBE buffer. The agarose buffer mixture was heated in the microwave for 1.5 min to melt the agarose. After cooling to 50–60 °C, 5 μL of SyberSafe (Cat: S33100, Thermo-Fisher) was added and mixed in well. The gel solution was carefully poured into the gel cast to avoid bubbles. The comb was placed at the appropriate position in the gel before it solidified, which typically took about 10 min. Two microliters of PCR product were mixed with 8 μL of sample dye and added to each well on the gel. The gel was run at 70 V in the running buffer for an hour.

### 4.6. Western blot analysis of wild-type and transgenic ΔN-MyBP-C rat heart fibers

The left ventricular tissue from ΔN-MyBP-C transgenic rats was dissected into small pieces and was digested in RIPA tissue buffer (Cat: R0278–50ML, Sigma-Aldrich) with a 0.1 % EDTA-free protease inhibitor cocktail. The tissue solution was incubated on ice for 10 min and tipsonicated on ice for a 10 s on/30 s off interval, repeated three to four times until no solid pieces remained in the solution. The sample solution was mixed with 2× SDS dye (Cat: LC2676, Novex) in a 1:1 ratio and heat-shocked at 95 °C for 5 to 10 min. The protein samples were run on a 9 % SDS-PAGE gel at 120 V for an hour. The samples on the SDS-PAGE gel were transferred to a PVDF membrane (Cat: LC2002, Thermofisher) using a semidry transfer method at 1.5 A for 30 min. The PVDF membrane with protein samples was incubated overnight with both C-terminal (Cat: PA5–79714, Thermofisher) and N-terminal MyBP-C antibody (Cat: sc-137180, Santa Cruz Animal Health), respectively, diluted to 1:1000. The western blot image was captured using the image machine and analyzed with ImageLab software.

### 4.7. Simultaneous measurements of isometric tension and FRET fluorescence intensity in skinned and reconstituted cardiac muscle fibers

To study the effects of sarcomere length on thin filament regulation, the force contraction and the steady-state FRET fluorescence intensity were measured during a calcium titration for both short and long sarcomeres. All the buffer recipes used in these experiments were from the previous paper from this lab [69]. Reconstituted cardiac muscle fibers were mounted between two clips inside the buffer chamber, which was prefilled with ice-cold pCa 9 buffer (50 mM BES, 5 mM NaN_3_, 2 mM EGTA, 5 mM NTA, 0.024 mM CaCl_2_, 6.87 mM MgCl_2_, 5.83 mM Na_2_ATP, and 71.14 mM K-propionate, 0.1 % protease inhibitor cocktails)) [70]. The positions of the fibers in the chamber were adjusted with tweezers and a microscope. One clip was connected to a 5-mN level force transducer (SI Heidelberg KG7A, WPI); the other clip was connected to a mechanical motor which can be adjusted manually. The fiber could be stretched to different sarcomere lengths using the mechanical motor, and the sarcomere length could be detected using a He–_Ne_ laser with a diffraction method. The entire fiber measurement system was kept at 4 °C by a circulatory cooling system (Cell MicroControls, CH). Calcium titration was generated by mixing the pCa 4.3 buffer (50 mM BES5 mM NaN_3_, 2 mM EGTA, 5 mM NTA, 10.11 mM CaCl_2_, 6.61 mM MgCl_2_, 5.83 mM Na_2_ATP, and 51 mM K-propionate, 0.1 % protease inhibitor cocktails) and pCa 9 buffer with a preset flow rate that continuously passed through the fiber chamber. Force contraction of the filaments was measured by the sensitive force transducer during the calcium titration. Following a previously established protocol in our lab [48], the changes in the FRET fluorescence intensity of cTnC(13C/51C)AEDENS-DDPM *vs*. Ca^2+^ titration were monitored at an emission peak of 510 nm with a 340-nm excitation from a LED source. The titration curves of changes in force tension or fluorescence intensity *vs*. Ca^2+^ concentration were analyzed using a modified Hill equation to derive pCa_50_ and the Hill coefficient

$$f=f_{0}+\frac{a{\left(10^{pCa}\right)}^{n}}{{\left(10^{pCa50}+10^{pCa}\right)}^{n}}$$

Here, *f* indicates the changes in normalized force or fluorescence intensity with pCa titration, and *f*_*0*_ is the minimal y value; a represents the normalized maximum *f*, and n is the Hill coefficient [71]. The accuracy of the free [Ca^2+^] measurement in the pCa titration was tested and calibrated using a Ca^2+^ chelator quin-2 solution. Quin-2 was used in this protocol to generate the correlation curve of Ca^2+^ concentration *versus* the pump flow rate of our titration system by measuring the fluorescence intensity of the titration solution. Quin-2 forms a stable fluorescent complex with Ca^2+^ but not Mg^2+^ and has an excitation wavelength at 339 nm and a peak emission wavelength at 490 nm. A 0.5 M stock solution of Quin-2 was made in 0.1 M Tris buffer, pH 7.4, and stored in the 4 °C fridge. First, different concentrations of [Ca^2+^], from 0.024 mM to 10.11 mM every 2 mM, were added to quin-2 solution and the fluorescence intensity was measured. Second, a Ca^2+^ titration with quin-2 buffer was applied with a certain pump flow rate in the titration system. The free [Ca^2+^] in the pCa buffer during titration was calculated and calibrated based on the paper. The modified equation of the pump flow rate was customized and put into the Excel sheet provided by the paper, and the free [Ca^2+^] in the pCa buffer titrated by our pump system could be calculated. Statistical significance is reported at *p* less than 0.05.

### 4.8. Skinned myofibril preparations and stopped-flow measurements

Myofibrils were prepared by following protocols adopted from the literature [51,72]. Briefly, fresh cardiac ventricle tissues from WT and transgenic ΔN-MyBP-C rats were cut into 1-mm-wide strips and washed 3× with fiber preparation buffer containing 6 mM imidazole, 8 mM Mg-acetate, 70 mM K-propionate, 5 mM EGTA, 7 mM ATP, and 1 mM NaN_3_ at pH 7.0 at room temperature. The washed strips were then skinned by being incubated in the fiber preparation buffer plus 0.5–1 % Triton X-100 and slowly rotated overnight at 4 °C. The skinned strips were transferred and washed with myofibril stopped-flow buffer containing: 106 mM K-acetate, 50 mM Imidazole, 12 mM Mg-acetate, and 2 mM EGTA at pH 7.0, followed by homogenization using a Dremel on ice at 15,000 rpm for 3x10s with 5-min intervals. The homogenized sample was then centrifuged at 2800 *g* for 10 min at 4° C. The pellet was then resuspended in fresh myofibril stopped-flow buffer. The suspended sample was centrifuged and resuspended an additional three times before the concentration of the myofibrils was measured using A_280_ with an extinction coefficient of 0.7 (mg/ml)^−1^ and blanking against the stopped-flow buffer; a reading of 1 at A_280_ equates to 140 nM myofibrils.

The stopped-flow experiments were performed with a KinTek stopped-flow spectrometer with the chamber temperature set to 20 °C for all experiments. The myofibril samples were incubated with 10 μM MANT-ATP for ~1 min. Experiments were conducted by rapidly mixing 250 μM ATP and the incubated 100 nM myofibrils. Listed concentrations of ATP and myofibrils are prior to mixing. Data were recorded on three scales, each with 1000 data points: 25, 200, and 500 s. Each full decay curve was reconstructed using all three sets of data obtained under the same conditions. Each constructed decay was fitted to a double-exponential function. The fast-decay component is associated with myosin molecules in the RDX state, while the long-decay component is associated with myosin heads in the SRX state [51–54]..

### 4.9. Statistical data analysis

The two sample *t*-test with assuming unequal variances in Excel was used to analyze the statistical significance level of the ΔpCa between each condition. The conditions that compared in the analysis include (a) wt-cTnI and ΔSP-cTnI, (b) wt-MyBP-C and ΔN-MyBP-C and (c) with and without MAVA drug. Comparisons were made to determine whether the differences of ΔpCa between each condition are by chance. Fitted values of ΔpCa are reported as mean ± SEM with statistical significance level set at *P < 0.05 or **P < 0.01.

## Figures and Tables

**Table 1:** Organization of pCa_50_, Hill coefficients (n), and ΔpCa_50_ values *vs*. Force and FRET for each condition.

Fiber	cTnI	XBs	Mava	Output	pCa_50_ (1.8)	nH	pCa_50_ (2.2)	nH	ΔpCa50
WT	WT	Yes	No	Force	5.77 ± 0.01	4.81 ± 0.011	5.98 ± 0.01	6.59 ± 0.012	0.21
	WT	Yes	No	FRET	5.74 ± 0.02	3.28 ± 0.014	5.95 ± 0.03	3.61 ± 0.025	0.21
			5 μM		5.61 ± 0.04	1.68 ± 0.021	5.72 ± 0.03	2.61 ± 0.017	0.11
	ΔSP	No	No	FRET	5.83 ± 0.03	1.75 ± 0.027	6.02 ± 0.04	1.82 ± 0.031	0.19
			5 μM		5.64 ± 0.04	2.26 ± 0.022	5.73 ± 0.03	2.47 ± 0.044	0.09
Fibers	cTnI	XBs	Mava	Output	pCa_50_ (1.8)	nH	pCa_50_ (2.2)	nH	ΔpCa50
	WT	Yes	No	Force	5.82 ± 0.01	3.91 ± 0.012	5.97 ± 0.01	6.59 ± 0.014	0.15
			No	FRET	5.64 ± 0.02	2.92 ± 0.016	5.78 ± 0.02	3.61 ± 0.025	0.14
ΔN	WT	Yes	5 μM		5.66 ± 0.03	2.21 ± 0.029	5.75 ± 0.03	2.86 ± 0.014	0.09
			No	FRET	5.72 ± 0.03	2.62 ± 0.027	5.86 ± 0.04	1.84 ± 0.021	0.14
	ΔSP	No	5 μM		5.66 ± 0.02	2.03 ± 0.026	5.74 ± 0.03	2.66 ± 0.032	0.08

**Table 2:** Ratio of SRX and DRX of TG and NTG myofibrils derived from stopped-flow experiments under various conditions.

Myofibril type	Condition	DRX %	SRX %
Wild-type (WT)	Control	53.1 ± 1.6	46.6 ± 1.6
	5 μM MAVA	38.5 ± 3.8	61.5 ± 3.8
ΔN-MyBP-C	Control	59.4 ± 4.6	40.6 ± 4.6
	5 μM MAVA	60.4 ± 0.8	39.6 ± 0.8
